# LC-QToF-Based Metabolomics Identifies Aberrant Tissue Metabolites Associated with a Higher-Fat Diet and Their ‘Reversion to Healthy’ with Dietary Probiotic Supplementation

**DOI:** 10.3390/metabo13030358

**Published:** 2023-02-28

**Authors:** Allyson Dailey, Gloria Solano-Aguilar, Joseph F. Urban, Robin D. Couch

**Affiliations:** 1 Department of Chemistry and Biochemistry, George Mason University, Manassas, VA 20110, USA; 2 Diet, Genomics, and Immunology Laboratory, Beltsville Human Nutrition Research Center, Agricultural Research Service, U.S. Department of Agriculture, Beltsville, MD 20705, USA

**Keywords:** probiotics, obesity, tissue metabolomics, untargeted metabolomics, metabolic reversion, probiotic-induced reversion, high-fat diet, healthy diet, LC-QToF, Ossabaw pigs

## Abstract

Over 33% of Americans are labeled as obese, leading the World Health Organization to designate obesity as a major public health problem. One consequence of obesity is the development of metabolic syndrome, a condition which has been correlated to an increased risk for developing cardiovascular disease and Type 2 diabetes. Prolonged ingestion of a higher-fat diet, one cause of obesity, results in alterations to the gut microbiome. These alterations are implicated to have a profound role in the evolution and progression of obesity-linked diseases. Probiotics are associated with positive health effects such as limiting pathogen colonization, aiding in digestion, and vitamin synthesis. Using Ossabaw pigs as a model for obesity, and in conjunction with our previous research, we performed an in-depth, nontargeted, metabolomic analysis on select organs to elucidate the effects of dietary supplementation with the probiotic *Lacticaseibacillus paracasei*. We focused our analysis on the effects of probiotic supplementation on a higher-fat (obesogenic) diet and a nutritionally balanced diet. Notably, our findings reveal that the brain cortex is highly sensitive to dietary influencers, and with probiotic supplementation, several aberrant metabolites associated with a higher-fat diet revert to healthy levels, thus demonstrating the potential for a probiotic intervention for obesity-linked disease.

## 1. Introduction

Probiotics are live microorganisms which resemble/reflect those found in the natural gut flora [1,2,3]. Probiotics, particularly from the genera *Bifidobacterium* and *Lactobacillus* (which presently is split over several new genera), have been associated with a number of health effects, including competitive exclusion of food pathogens, stimulation of immune function, lowering gas distension, aiding in food digestion and adsorption, synthesis of vitamins, lowering cholesterol levels, and may also aid in the curing of gastrointestinal diseases and autoimmune disorders [1,4,5,6,7,8,9,10,11,12,13,14]. While few studies have yet addressed the potential of probiotics for the management of obesity, a higher-fat diet is known to induce diabetes and endotoxemia in mice, and negatively correlates with the level of *Bifidobacterium* spp. in the mouse intestine [15]. By supplementing these mice with the prebiotic oligofructose, enhanced growth of intestinal *Bifidobacterium* occurs with accompanying normalization of the inflammatory state (decreased endotoxemia, decreased plasma and adipose tissue pro-inflammatory cytokines), suggesting that *Bifidobacterium* in the gut microbiota may prevent the deleterious effects of higher-fat diet-induced metabolic disease. Furthermore, a selected strain of *Lacticaseibacillus rhamnossus* has also been reported to protect mice from diet-induced obesity, likely due to the production of conjugated linoleic acid by the bacteria [16]. Even with the increasing accumulation of clinical data on the benefits of pre- and probiotics, the mechanism of modulation remains largely uncharacterized, especially with respect to the host metabolome. Hence, to further explore the effect of a higher-fat diet and the consequences of probiotic supplementation, this investigation examined pig tissue metabolomes through non-targeted metabolomic profiling.

Given their anatomical and metabolic similarities to humans, Ossabaw pigs were selected for use as a model organism in this study. Previously, we reported that a higher-fat diet alters the metabolic composition of select Ossabaw pig tissues, relative to tissues in pigs fed a nutritionally balanced diet [17]. Following this report, we examined the alterations to the serum, urine, and fecal metabolomes of conventional pigs as a consequence of a higher-fat, higher cholesterol diet, relative to a nutritionally balanced diet [18]. The results of both studies revealed that bile acids, lipids, amino acids, phosphatidic acid, phosphatidylcholines, and glycerophospholipids are particularly sensitive to the dietary change. Finally, we previously examined and reported the effect of probiotics on pig cholesterol metabolism and inflammation [19]. The results of this study showed that when coupled to a higher-fat diet, probiotic supplementation modulates cholesterol metabolism and reduces the inflammation observed within the higher-fat cohort. Thus, to complement the results from the previous studies, our current investigation sought to determine if dietary supplementation with the probiotic *Lacticaseibacillus paracasei* subsp. *paracasei* L. casei W8 also has a measurable effect on the host tissue metabolism. To accomplish this, we specifically compared metabolomic profiles from select organ tissues, obtained from pigs fed either a nutritionally balanced (basal) or obesogenic (higher-fat) diet, with or without probiotic supplementation.

## 2. Materials and Methods

### 2.1. Maintenance of Animals and Experimentation

Ossabaw pigs (born at the Indiana University Ossabaw Production Unit) were transported in kennels and delivered overnight to the USDA-Beltsville Maryland animal facility according to standardized procedures of quarantine, and under the approval of the Beltsville Area Animal Care and Use Committee (protocol number: 09-017). After arrival, the pigs were housed in individual pens (within an isolated building) with free access to water, and fed a standard mini pig grower diet (5L80 Purina TestDiet, Inc., Richmond, IN, USA) specifically designed for miniature swine (18.5% kcal from protein, 71.0% from carbohydrates and 10.5% from fat) [20]. After acclimatization, all eight-week-old pigs were randomized by weight and split into four treatment groups. Pigs in Cohorts 1 (n = 5) and 3 (n = 5) continued eating the mini pig grower diet 5L80 (basal diet), with gradual bi-weekly step increases from 750 to 3600 kcal/day, to adjust for nutrient requirements for their growth during the 24 weeks of the study. A standard, higher-fat (obesogenic) pig diet was given to the other two treatment groups (Cohorts 2 (n = 5) and 4 (n = 5)). This diet was prepared at the Beltsville feed mill by mixing a commercial diet (5KA6 Purina TestDiet, Inc, Richmond, IN, USA) with 17% hydrogenated soybean oil, containing 56% trans fatty acids (#170, Columbus Foods, Chicago, IL, USA), 2.4% corn oil, 1% cholesterol, 0.7% cholic acid, and recommended levels of minerals and vitamins for swine. This mixture yields 13% of total kcal from protein, 57% kcal from carbohydrates, and 30% kcal from fat. The pigs on the higher-fat diet received bi-weekly step increases from 2000 to 4500 kcal/day for the duration of the study. Daily food rations were pre-weighed, and consumption was individually monitored and recorded daily. As previously described, starting at week 8, all dietary groups were supplemented daily with an oral gavage of either the probiotic bacteria (*L. paracasei* subsp. *paracasei* L. casei W8; 1 × 10^10^ cfu/day (a probiotic previously shown to modify triacylglycerol levels and suppress energy intake)) (Chr. Hansen, Hoersholm, Denmark) (Cohorts 3 and 4) or an equivalent volume of a probiotic-free maltodextrin vehicle (placebo), dissolved in 5 mL of phosphate-buffered saline (PBS) solution (Cohorts 1 and 2) [19,21,22]. Aliquots of the lyophilized probiotic and placebo provided by the manufacturer (Chr. Hansen) were cultured to adjust the probiotic cfu dosage and to verify their viability. At the completion of the study, each pig was euthanized using the standard approach (500 mg ketamine (Ketaset, Fort Dodge Animal Health, Fort Dodge, IA, USA), 80 mg tiletamine (Telazol, Fort Dodge Animal Health, Fort Dodge, IA, USA), 80 mg zolazepam (Telazol), and 333 mg xylazine (Xyla-Ject, Phoenix Pharmaceutical, St. Joseph, MO, USA), per 100 kg body weight). Immediately following euthanasia, the tissue samples (brain cortex, heart, kidney, liver, skeletal muscle, and pancreas) were collected, snap-frozen in liquid nitrogen, and stored at −80 °C. All animal experiments and procedures were conducted in accordance with guidelines established and approved by the Beltsville Area Animal Care and Use Committee.

### 2.2. Sample Preparation for Metabolite Profiling

While using liquid nitrogen to keep the tissue samples frozen, the samples were ground into fine powder (TissueLyser II, Qiagen, Germantown, MD, USA), and then weighed into pre-chilled microcentrifuge tubes (∼100 mg aliquots). The powdered tissues were then extracted using a methanol:water solution (1:1), added in a ratio of 3 μL of solvent/mg frozen tissue. The sample was vortexed, sonicated at room temperature for 5 min, clarified by centrifugation (15 min at 16.1 rcf), and the supernatant was collected and stored on ice until it was analyzed [17].

### 2.3. LC-QToF Analysis

Samples were analyzed via an Agilent 1290 Infinity UPLC coupled to an Agilent 6450 Accurate Mass QToF (Agilent, Santa Clara, CA, USA), running in ESI positive and negative modes. Analyte separation was performed by the LC-MS metabolomics facility at the University of Eastern Finland, using two different chromatographic systems to gain wide coverage of metabolites (BEH Amide HILIC column (Waters, Milford, MA, USA), as well as a C18 reversed phase column (Agilent, Santa Clara, CA, USA) [23,24]. A quality control (QC) sample was prepared by pooling a small aliquot of each sample to represent the total metabolite composition present in the analysis. This QC sample was injected after every 12 randomized analytical samples to serve as a monitor for potential problems in the chromatography and/or in ion response. For each tissue (using a small aliquot pool of all 20 samples in that tissue type), MS/MS was performed using collision energies of 10 eV, 20 eV, and 40 eV.

### 2.4. Data Processing, Chemometrics, and Statistical Analysis

Molecular features were identified in the raw chromatograms using Agilent Technologies’ MassHunter Qualitative Analysis software (ver. B.06.00, Agilent, Santa Clara, CA, USA). The molecular features and their relative abundance (peak height) were then tabulated using Agilent Technologies’ Mass Profiler Professional software (ver. 12.6, Agilent, Santa Clara, CA, USA), resulting in a metabolomic dataset containing the molecular features identified by HILIC ESI positive, HILIC ESI negative, reverse phase ESI positive, and reverse phase ESI negative modes. Metabolites present in ≤19% of the total number of samples processed were treated as one-offs and were removed from the matrix. For subsequent data processing, the samples in the metabolite matrix were organized by their appropriate cohort (a nutritionally balanced basal cohort, a higher-fat obesogenic cohort, a basal cohort supplemented with probiotics, and a higher-fat cohort supplemented with probiotics), and the data was further filtered to include only those analytes that were present in at least 3 of 5 pigs for any 1 cohort. Next, in each cohort, the outlier peak height values were identified using an analysis of (cohort mean-cohort median)/cohort median for each metabolite, and a cutoff value ≥ 1.5 [25]. Outliers were replaced with the median cohort value for that metabolite. Metabolite peak height values were then standardized across the four cohorts via their conversion to Z-scores. A principal component analysis was then performed in R using the standardized metabolite matrices, and the results were subsequently visualized using the package rgl. Microsoft Excel was used to perform the fold-change calculations, the two-sample *t*-tests, and to obtain the Benjamini–Hochberg critical values (to adjust for the false discovery rate). Volcano plots were generated using the R statistical package EnhancedVolcano. Pearson’s correlation coefficients were calculated using Microsoft Excel, and a correlation network was created using the R statistical package qgraph. Finally, for the molecular features exhibiting the greatest statistical difference, extracted ion chromatograms (EICs) were generated using Agilent’s Mass Hunter Qualitative Analysis software. Features were deemed significant when they appeared as a single symmetrical peak at the expected retention time. Only significant molecular features were considered in this investigation. For these features, the MS/MS data was analyzed by comparing the fragmentation profiles to known compounds in either Metlin (The Scripps Research Institute, La Jolla, CA, USA, the Human Metabolome Database (HMDB, University of Alberta, Edmonton, AB, Canada), MassBank of North America (MoNA, UC Davis West Coast Metabolomics Center, Davis, CA, USA), or NIST17 (NIST, Gaithersburg, MD, USA). Features which were unidentified by the databases were analyzed using MS-FINDER (UC Davis West Coast Metabolomics Center, Davis, CA, USA) to elucidate chemical formulas, and then, based on those formulas, in silico MS/MS matching was performed to provide metabolites’ names.

## 3. Results and Discussion

### 3.1. Dietary Effects on the Pig Tissue Metabolomes

**Figure 1:**
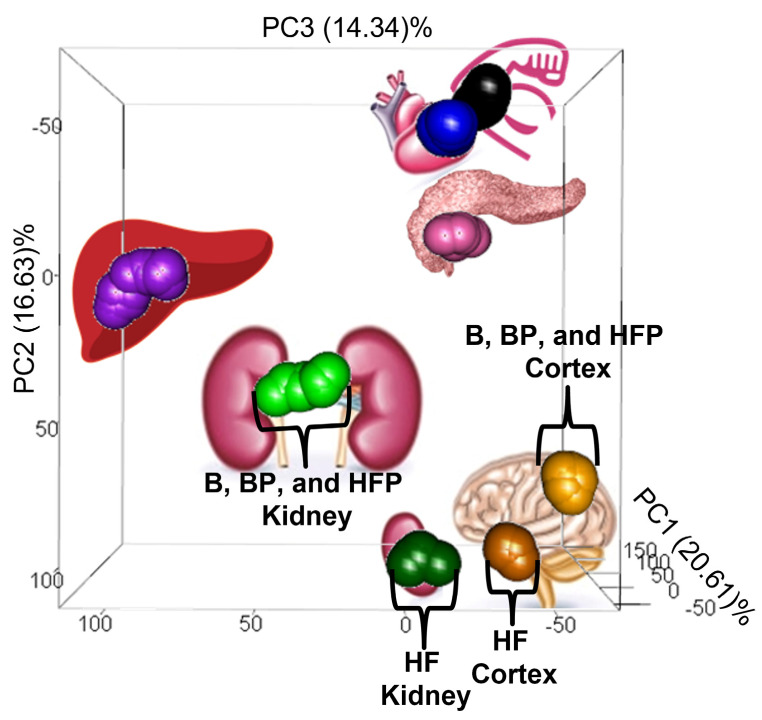
PCA plot of all derived pig tissue metabolomes. A total of 20 pigs were distributed equally into 4 distinct dietary cohorts (a nutritionally balanced basal cohort, a higher-fat obesogenic cohort, a basal cohort supplemented with probiotics, and a higher-fat cohort supplemented with probiotics). A PCA compared the metabolomes, and a three-dimensional plot of the results reveals how the pig tissue metabolomes are distinguished from each other (that is, the samples distinctly cluster by tissue type in the plot). Each sphere in the plot reflects the metabolome of one pig tissue sample and is color-coded by tissue type (liver: purple, kidney: green, heart: blue, skeletal muscle: black, pancreas: pink, and brain cortex: orange). For emphasis, the kidney samples obtained from the higher-fat pig cohort are colored dark green, and the higher-fat brain cortex samples are colored dark orange (they are also denoted as HF in the plot). This PCA plot demonstrates that, regardless of dietary cohort, the heart, skeletal muscle, pancreas, and liver samples cluster by tissue type. In contrast, the kidney and brain cortex samples obtained from pigs fed a higher-fat diet segregate away from the same tissues obtained from the basal, basal+probiotic, and higher-fat+probiotic cohorts (for emphasis, these are labeled as B, BP, and HFP in the plot), suggesting that these tissues undergo a more significant change in response to a higher-fat diet. These changes, however, appear to revert with probiotic supplementation.

A total of 20 pigs were distributed equally into four distinct dietary cohorts: (1) those fed a nutritionally balanced basal diet (378 kcal from fat, 2556 kcal from carbohydrates, and 666 kcal from protein per day); (2) those fed a higher-fat obesogenic diet (1350 kcal from fat, 2565 kcal from carbohydrates, and 585 kcal from protein per day); (3) those fed a basal diet supplemented with probiotics (*L. paracasei* subsp. *paracasei* and L. casei W8; 1 × 10^10^ cfu/day); and (4) those fed a higher-fat diet supplemented with probiotics (*L. paracasei* subsp. *paracasei* and L. casei W8; 1 × 10^10^ cfu/day). From each pig in each cohort, tissue samples (brain cortex, heart, kidney, liver, skeletal muscle, and pancreas) were collected, and their metabolomes were analyzed via LC-QToF under reverse phase and HILIC chromatographic conditions, in ESI positive and negative modes. Following data collection and processing, a principal component analysis (PCA) of all samples was performed. The resulting PCA plot, shown in Figure 1, depicts clear metabolic differences between the pig organs. Overall, while the differences among the various tissue types are made apparent by the plot, it is interesting to note that the heart and skeletal muscle appear to be closely juxtaposed in the plot, a reflection of their greater similarity in metabolome composition compared to the other tissues. Additionally, the PCA plot suggests that the kidney and brain cortex tissues are particularly sensitive to a higher-fat diet (i.e., the metabolomes derived from the higher-fat pig kidney and brain cortex samples segregate from those obtained from the same tissues in pigs fed either a basal, basal+probiotic, or higher-fat+probiotic diet). Furthermore, based upon this clustering pattern, probiotic supplementation to the higher-fat diet appears to alter the higher-fat metabolomes (i.e., the higher-fat+probiotic samples cluster tightly with the basal and basal+probiotic samples).

### 3.2. Metabolites Altered Due to a Higher-Fat Diet

**Figure 2:**
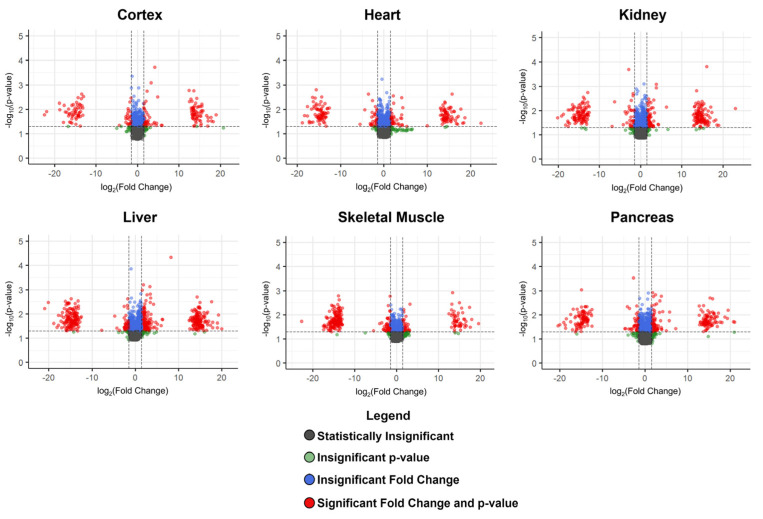
Tissue-specific volcano plots comparing the basal versus higher-fat diet cohorts. Within each identified tissue type, the fold-change in metabolite abundance is calculated as the ratio of the median chromatographic peak height in the higher-fat cohort relative to the corresponding median peak height in the basal cohort. In the volcano plots, the –log_10_ of the Benjamini–Hochberg-corrected *p*-value for each metabolite is presented as a function of the log_2_ of the relative abundance fold change. A Benjamini–Hochberg critical cutoff value of 0.05 was used to perform the *p*-value corrections. The horizontal dashed line in the plots identifies a corrected *p*-value of 0.05 (i.e., –log_10_(*p*-value) = 1.3), while the vertical dashed lines denote a log_2_ fold-change of 1.5 in metabolite abundance (increase and decrease). Each metabolite is represented as a circle in the plot and color-coded as per the figure legend.

While the brain cortex and kidney are the most susceptible to higher-fat induced dietary changes (Figure 1), higher-fat diet associated alterations were observed among the other pig tissues analyzed. To identify those metabolites which were greatly influenced by a higher-fat diet, we compared the derived metabolomes of the pigs fed a basal and higher-fat diet for each tissue type. Within every tissue examined, numerous metabolites were found to be either unaffected, significantly increased in abundance, or significantly decreased by a higher-fat diet (Figure 2).

To identify the top metabolites that are greatly influenced by a higher-fat diet, we focused our attention on the tissue features that were present in at least 60% of any one cohort (i.e., the affected tissue metabolite was identified in at least three of the five pigs, in any one of the two compared cohorts), and ranked these metabolites by fold-change, *p*-value, and quality of the extracted ion chromatogram (EIC). All metabolites presented in Table 1 were putatively identified via MS/MS fragmentation (10 eV, 20 eV, and 40 eV for each metabolite), by matching the corresponding fragmentation patterns to known compounds in either Metlin, the Human Metabolome Database (HMDB), MassBank of North America (MoNA), or NIST17. These metabolites and their relationship to a higher-fat diet are discussed in further detail below.

#### 3.2.1. Diet-Associated Alterations in the Brain Cortex 

As indicated in Figure 1, the brain cortex is one of the tissues most susceptible to a higher-fat diet. Though many metabolites contribute to the differences, glutathione and S-adenosylhomocysteine were highlighted as major contributors (Table 1). Glutathione fell below the limit of detection in the brain cortex tissue obtained from the higher-fat cohort of pigs, thus depicting a higher-fat diet-induced suppression of glutathione abundance, relative to basal-diet-fed pigs. Glutathione is a tripeptide molecule which protects against reactive oxygen species (ROS), thereby promoting healthy neuronal function and preventing neurological diseases [26,27,28,29]. This higher-fat diet-associated reduction in the level of glutathione has also been observed in mice, supporting the notion that a higher-fat diet induces alterations to glutathione metabolism, which in turn may lead to neurocognitive decline [28,29,30,31].

A higher-fat diet also was found to increase the intracellular concentration of S-adenosylhomocysteine (SAH) in the brain cortex. SAH is part of the methyl cycle, and is derived from S-adenosylmethionine (SAM), a methylating agent found in numerous biosynthetic reactions (catalyzed by a variety of methyltransferases) [32,33,34]. SAH is known to act as a negative regulator of several of these methyltransferases, thereby inhibiting the rate of methylation using SAM [35]. Studies have shown that an elevated SAH concentration is correlated to DNA hypomethylation, leading to an increase in ROS, and in turn, an inhibition of the methylation of tRNAs responsible for translating the selenoproteins, which normally reduce H_2_O_2_ levels [35]. Additionally, elevated SAH has been linked to an increase in cognitive impairment and accelerated disease progression in Alzheimer’s patients [36,37]. Thus, based on the evidence of increased SAH and decreased glutathione within the brain cortex of higher-fat pigs, it is interesting to speculate that prolonged consumption of a higher-fat diet may lead to neurocognitive decline.

#### 3.2.2. Diet-Associated Alterations in the Heart and Pancreas

Within the heart and the pancreas, the higher-fat cohort of pigs have a lower abundance of betaine, lower levels of trimethylamine n-oxide (TMAO) in the pancreas, and lower levels of arabitol in the heart, relative to their basal diet cohort (Table 1). Obtained via choline oxidation or dietary supplementation, betaine is an osmoprotectant, antioxidant, and methyl donor that participates in the conversion of homocysteine to methionine, an essential step in the synthesis of SAM [38,39]. Reduced amounts of betaine can result in the accumulation of homocysteine, which in turn can lead to cardiovascular disease [40]. Similarly, TMAO is an oxidation product of trimethylamine, a gut microbiome metabolite derived from either betaine, choline, or carnitine [41,42,43]. While an elevation in circulating TMAO has been linked to an increase in cardiovascular disease, this does not necessarily reflect its metabolic effect within the tissues [44]. For example, within the pancreas, TMAO supplementation has been shown to improve islet cell impairment in Type 2 diabetes [44]. Hence, the reduction of TMAO within the pancreas of the higher-fat cohort of pigs may reflect altered microbiome activity and a reduction in islet cell functionality. Finally, we compared the choline levels within the basal feedstock and higher-fat feedstock (as the concentration of betaine within the feed is unknown), and found only a moderately lower abundance in the higher-fat feedstock (1500 ppm versus 1200 ppm, respectively [20]).

As mentioned above, within the heart of the higher-fat cohort of pigs, the level of arabitol was found to be low, relative to the basal cohort. While the reduction in arabitol is unclear, arabitol is a sugar alcohol known to be synthesized by commensal osmophilic fungi (to regulate osmotic pressure), and via xylulose it can be routed into the non-oxidative phase of the pentose phosphate pathway (PPP) [45,46,47,48]. As a higher-fat diet is known to alter the gut mycobiome, perhaps the suppression of arabitol is due to altered fungal composition, and in turn, reduced flux through the PPP within the heart of the higher-fat cohort of pigs [49,50]. Since the PPP is important in maintaining cellular redox and assisting in cardiomyocyte proliferation, a reduction in the levels of the intermediates involved in the PPP may lead to cardiovascular disease [51].

#### 3.2.3. Diet-Associated Alterations in the Kidney 

Indoxyl sulfate (also referred to as indican) was found to be suppressed in the kidney of the higher-fat cohort (Table 1). Indoxyl sulfate is a product of tryptophan degradation and protein putrefaction [52,53]. Within the intestines, tryptophan is converted by bacteria into indole, which can be absorbed into the blood stream and then further metabolized in the liver. Because it can disrupt membrane integrity, indole accumulation is toxic [54]. Thus, indole is subsequently converted by the liver into indoxyl sulfate, which migrates to the kidneys and is ultimately excreted in the urine [53]. While a healthy (basal) diet led to measurable levels of indoxyl sulfate in kidney tissue, kidneys obtained from pigs fed a higher-fat diet were found to be devoid of this metabolite. While the reason for this is unclear, we speculate that the gut microbiome is preferentially utilizing the abundance of fat for fuel, rather than protein degradation/amino acid oxidation.

#### 3.2.4. A Reduction in the Membrane PC/PE Ratio

In agreement with previous findings [17], within several of the examined tissues, the abundance of phosphatidylcholine (PC) and phosphatidylethanolamine (PE) is altered with a higher-fat diet. PCs and PEs are the two most abundant phospholipids within mammalian cell membranes [55]. PCs are located primarily in the outermost sheet of the lipid bilayer, whereas PEs predominate in the inner sheet. Research has shown that dietary oils can have a measurable effect on membrane lipid composition, and the PC/PE ratio is considered to be an important marker of liver health [56]. A reduction in this ratio reflects the accumulation of PE within the hepatic membrane, thereby altering membrane fluidity, and has been found to contribute towards non-alcoholic fatty liver disease (NAFLD) [55]. Accordingly, while alterations to the hepatic PC/PE ratio are linked to liver impairment, PC supplementation has been shown to improve overall liver function in NAFLD patients [55,57]. With respect to the liver tissue obtained from the higher-fat cohort of pigs, our study identified a reduction in PC and elevation in PE, supporting the notion that a higher-fat diet can lead to altered membrane fluidity and perhaps, ultimately, hepatic impairment. Further, PE elevation was also found within the muscle, pancreas, and kidney tissues obtained from pigs in the higher-fat diet cohort. Elevated PE has been described in chronic kidney disease [58]. As a higher-fat diet is linked to renal injury, perhaps altered renal membrane fluidity plays a contributing role in the etiology of the disease [59].

#### 3.2.5. Diet-Associated Alterations in the Liver 

Bile acids are fat emulsifiers, facilitating the absorption of lipids and fat-soluble vitamins within the small intestine. Additionally, bile acids are known to act as signaling molecules, activating G-protein-coupled receptors (GPCRs) and nuclear receptors to regulate energy metabolism, inflammation, and cell death [60,61,62,63]. Within the liver, increased secretion of the bile acid glycocholic acid (a cholic acid derivative) was noted in the higher-fat cohort of pigs (Table 1). Prolonged consumption of a higher-fat diet has been to shown to increase bile acid secretion (i.e., glycocholic acid), ultimately leading to chronic liver diseases [64,65,66]. Thus, we postulate that a higher-fat diet upregulates bile acid synthesis, which, in turn, which may eventually lead to reduced hepatic function.

#### 3.2.6. Diet-Associated Alterations in the Skeletal Muscle 

The metabolite 7α-hydroxy-3-oxo-4-cholestenoic acid, a degradation product of cholesterol and precursor to chenodeoxycholic acid, was found to be elevated in the skeletal muscle of pigs fed a higher-fat diet [67,68]. Intriguingly, an accumulation of chenodeoxycholic acid has been shown to induce skeletal muscle atrophy [65]. Additionally, our investigation revealed that a higher-fat diet leads to an accumulation of uridine diphosphate glucuronic acid (UDP-GlcA) within the skeletal muscle. UDP-GlcA serves many roles as a precursor molecule in sugar nucleotide biosynthesis and as a cofactor in glucuronidation [69]. Catalyzed by a family of uridine diphosphoglucuronate glucuronosyltransferases (UGT), glucuronidation is required for the detoxification of a variety of endogenous and exogenous metabolites [70,71]. Previous studies have correlated a higher-fat diet with reduced UGT transcription, and demonstrated that free fatty acids can act as inhibitors to various isoforms in the UGT family [72,73,74]. While these findings provide evidence that a higher-fat diet may lead to a suppression in glucuronidation and, subsequently, detoxification, further research is required to determine if the UDP-GlcA accumulation observed in our study is a direct result of UGT inhibition and/or repression.

Finally, we discovered that within the skeletal muscle, a higher-fat diet leads to an accumulation of inosine, a byproduct of purine degradation. While the exact cause of the accumulation is unclear, an increased degradation of adenosine has been shown to occur in muscular atrophy [75]. Thus, given the accumulation of inosine and 7α-hydroxy-3-oxo-4-cholestenoic acid, we can speculate that prolonged consumption of a higher-fat diet may lead to muscular atrophy.

Overall, we see that consumption of the higher-fat diet leads to notable changes in tissue-associated metabolites. We next sought to determine if probiotic supplementation has any impact on those changes.

### 3.3. Metabolites Altered Due to Probiotic Supplementation

As noted in Figure 1, the kidney and brain cortex metabolomes derived from pigs fed a higher-fat diet segregate away from the metabolomes derived from the same tissues, obtained from the basal, basal+probiotic, and higher-fat+probiotic cohorts, yet these changes appear to revert with probiotic supplementation. Thus, to identify probiotic-associated changes, we compared the derived tissue metabolomes obtained from the basal versus basal+probiotic and higher-fat versus higher-fat+probiotic cohorts. The results of these analyses are tabulated in Table 2 below.

As visualized in Table 2 above, probiotic supplementation to the basal and higher-fat diet has a measurable effect on every tissue examined. Though the rationale behind many of these alterations is unclear, there are a few noteworthy metabolites and metabolic families (for example, phosphatidylcholines) that are described in further detail below.

#### 3.3.1. Phosphatidylcholines

When comparing the basal and higher-fat diets to their respective probiotic supplemented cohorts, we noticed that within the brain cortex, liver, and skeletal muscle, probiotic supplementation to both a basal and higher-fat diet reduces the levels of phosphatidylcholine (PC) (described previously in Section 3.2: *Metabolites altered due to a higher-fat diet*). Although the mechanism of suppression of PC within our probiotic supplemented cohorts is unclear, within NAFLD induced mice, supplementing a higher-fat diet with *L. paracasei* in a postbiotic fashion has been shown to reduce PC, suppress hepatic inflammation, and alter the gut microbiota composition [76]. Thus, while the underlying biochemical mechanisms of the reduction in PC is poorly understood, we can speculate that perhaps it may reflect a change in microbiome composition, which, in turn, may protect against metabolic diseases.

#### 3.3.2. Uridine Diphosphate-N-Acetylglucosamine

Within the kidney of Ossabaw pigs, uridine diphosphate-n-acetylglucosamine (commonly referred to as UDP-GlcNAc) was among the identified metabolites greatly influenced by probiotic supplementation (Table 2). In fact, UDP-GlcNAc was undetected in all five of the kidney samples obtained from the basal+probiotic cohort pigs, while it was abundant in four of the five kidney samples obtained from the basal diet cohort of pigs. This phenomenon was also observed when we compared the higher-fat cohort (detected in three out of the five pigs) to the higher-fat+probiotic cohort (undetected in all five pigs), again underscoring the correlation between probiotic supplementation and lowered UDP-GlcNAc abundance in the kidney. Synthesized de novo from fructose 6-phosphate and glucosamine via the hexosamine biosynthetic pathway, UDP-GlcNAc is utilized by the Golgi apparatus for O-linked protein glycosylation [77,78,79,80,81]. Metabolic flux through the hexosamine biosynthetic pathway is a reflection of nutrient availability, particularly that of glucose, glucosamine, UDP, and acetyl-CoA [79]. Since 3–5% of glucose is typically destined for UDP-GlcNAc production, probiotic supplementation may alter the availability of these nutrients, thereby affecting the intracellular UDP-GlcNAc concentration [77,78]. This probiotic-associated decrease in UDP-GlcNAc is unique to the kidney tissue, as it was not observed in any of the other tissues we analyzed (i.e., the UDP-GlcNAc level was consistent among the cohorts in these other tissues). While the mechanism of probiotic-associated suppression remains unclear, studies have shown that O-linked glycosylation is positively regulated by the intracellular concentration of UDP-GlcNAc, suggesting that a consequence of low levels of UDP-GlcNAc would be altered glycoprotein production within the basal+probiotic and higher-fat+probiotic pigs kidney cells [81]. Interestingly, increased O-linked glycosylation of proteins has been implicated in insulin resistance, chronic kidney disease, and obesity [82,83]. It remains unknown if probiotic supplementation might then serve to reduce the risk of metabolic syndrome, by reducing intracellular kidney UDP-GlcNAc levels.

#### 3.3.3. Saccharopine

Our investigation also discovered that within the liver, probiotic supplementation to the basal diet leads to significantly decreased amounts of saccharopine. While four of the five liver tissues obtained from the basal diet pig cohort have abundant saccharopine, it was below the limit of detection in all the basal+probiotic pig liver samples. Saccharopine is an intermediate within the lysine catabolic pathway in the liver [84,85,86,87]. Unlike other amino acids, lysine does not undergo direct transamination in amino acid oxidation. Rather, lysine and α-ketoglutarate condense to form saccharopine, which is subsequently oxidized to form α-aminoadipic δ-semialdehyde and glutamate via the bifunctional enzyme aminoadipic semialdehyde synthase [84,85,88]. Downstream metabolic pathways can route α-aminoadipic δ-semialdehyde and glutamate for nitrogenous biomolecule biosynthesis and energy production in the liver, so it is tempting to suppose that with the basal diet, the pigs are utilizing dietary lysine as an energy source, but with probiotic supplementation, the added probiotic bacteria utilize the lysine pool in the GI tract, thereby lowering the saccharopine concentration within the liver. Notably, the liver saccharopine concentration remained relatively consistent between the higher-fat and higher-fat+probiotics cohorts (fold change = −1.28), suggesting that the caloric abundance of the higher-fat diet does not necessitate amino acid oxidation by the probiotic bacteria (or the liver). While the cause of the reduced liver saccharopine concentration in the basal+probiotic cohort remains speculative, perhaps the significance/effect of the reduced saccharopine concentration in the liver is more important, which requires further investigation.

Finally, probiotic supplementation altered S-adenosylhomocysteine, indoxyl sulfate, and inosine levels (described previously in Section 3.2: *Metabolites altered due to a higher-fat diet*). When examining the higher-fat versus higher-fat+probiotic dietary cohorts, we saw that probiotic supplementation altered the levels of these three metabolites to a metabolic profile resembling the basal diet (i.e., if the metabolite was suppressed in the basal cohort, it was also suppressed in the higher-fat+probiotic cohort). These incidences of probiotic-induced metabolic reversions prompted us to further explore if probiotic supplementation can alter a higher-fat metabolome to one akin to a basal diet.

### 3.4. Probiotic-Induced Metabolic Reversions

**Figure 3:**
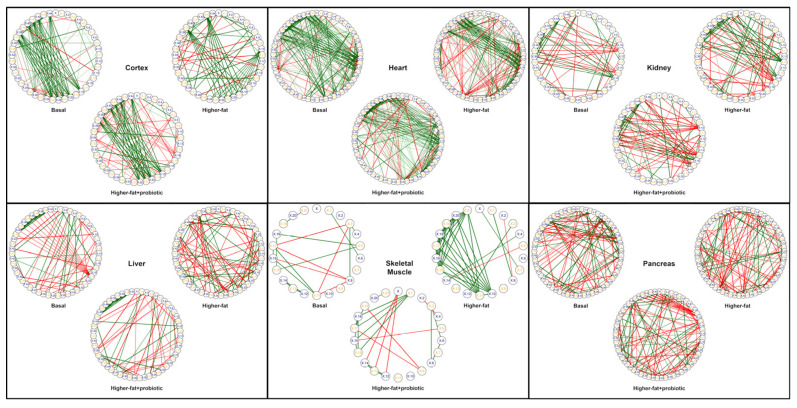
Tissue-specific metabolite correlation networks of the basal, higher-fat, and higher-fat+probiotic cohorts. For each tissue type, Pearson’s correlation coefficients were calculated for metabolites present in a minimum of 3 of 5 pigs in any one of the three pig cohorts, and only those metabolites common to all three cohorts were included in the network. Positive Pearson correlation values, r ≥ 0.90, are depicted as a green line between metabolites, whereas negative correlations, r ≤ −0.90, are depicted as a red line between metabolites. To facilitate visual comparison of the networks, metabolites are represented as a small, alpha-numerically labeled circle, and their placement around the circumference of each network is fixed among the plots for each tissue. For all tissues, comparison of the metabolite correlation networks (i.e., metabotype) indicates that probiotic supplementation of the higher-fat diet alters the metabolome relative to the higher-fat diet alone, and particularly within the brain cortex, heart, and kidney, probiotic supplementation changes the higher-fat metabolome in a manner that appears similar to the metabotype associated with the basal diet.

As illustrated in Figure 3, we next compared the derived tissue metabolomes via a series of network correlation maps, which depict the relationship among metabolites within the tissues (i.e., the metabotype). As seen in the Figure and in agreement with our results presented in Section 3.2, for every tissue examined, the higher-fat metabotype is visually distinct from the basal diet metabotype. Further, dietary probiotic supplementation has a profound impact on the tissue metabotype, changing the appearance of the correlation maps relative to those associated with the higher-fat diet. Intriguingly, for many tissue metabotypes (most notably in the brain cortex), probiotic supplementation of the higher-fat diet leads to alterations in the metabolite network, such that they assume a metabotype more similar to that associated with the basal diet (i.e., while the network correlation map of the higher-fat cohort is visually different than that of the basal cohort, supplementation with probiotics alters the higher-fat metabolome such that it appears more akin to that seen with the basal cohort). That is to say, for several tissues (brain cortex, heart, and kidney), probiotic supplementation appears to revert the higher-fat-associated metabotype to one more comparable to a nutritionally balanced, basal diet (even though the pigs continued to consume an obesogenic diet).

To examine this reversion phenomenon in more detail, we focused our attention on the brain cortex, heart, and kidney tissues, and examined those metabolites which differed between a basal and higher-fat diet. Using this filtered list of metabolites, we next looked at those metabolites which remained consistent between the basal and higher-fat+probiotic cohorts. Listed in Table 3 are the higher-fat-associated metabolites that revert to a “basal-like” level with probiotic supplementation. Of these 10 metabolites, 3 could be successfully identified via MS/MS by fragmentation pattern matching to known compounds. These three compounds were previously identified and described in Table 1 above (see Section 3.2: *Metabolites altered due to a higher-fat diet*). The remaining seven metabolites were identified using MS-FINDER, as described in the Materials and Method section. Interestingly, the brain cortex once again appears to be greatly influenced by the diet and is particularly sensitive to probiotic supplementation. In other words, not only did this tissue exhibit the most visually compelling evidence of probiotic-induced metabolic reversions in Figure 3, but it had the highest number of features implicated in this reversion.

#### 3.4.1. Probiotic-Induced Reversion in the Brain Cortex

In our investigation, the brain cortex appears to be the tissue most susceptible to dietary-induced metabolic alterations. Within the brain cortex, a higher-fat diet results in an increase in the intracellular concentration of S-adenosylhomocysteine (SAH). However, when this diet is supplemented with probiotics, SAH levels are suppressed, thus reverting the metabolite to a phenotype more akin to one associated with a healthy basal diet. As described previously, an increased concentration of SAH is correlated with cognitive decline, and thus while the mechanism underlying the phenomenon remains unknown, the effect of suppressing the intracellular SAH abundance via probiotic supplementation is highly desirable [36,37]. Additionally, probiotic supplementation to a higher-fat diet reverted glutathione to a basal level, thus providing additional evidence that probiotic supplementation may offset the metabolite markers of neurocognitive decline visualized in the higher-fat cohort.

#### 3.4.2. Probiotic-Induced Reversion in the Heart

The lysophospholipid LysoPE (18:2) was elevated within the heart of the higher-fat cohort of pigs and subsequently suppressed upon probiotic supplementation (resulting in a phenotype akin to the basal cohort). As a family, lysophospholipids play a role as precursors for membrane biosynthesis and as signaling molecules, promoting plaque formation, which leads to atherosclerosis [89,90,91,92,93]. LysoPE (18:2) in particular, has been shown to induce lipid droplet formation and accumulation (within hepatocytes) and is reported in cases of non-ST-elevation myocardial infarction (NSTEMI) [94,95]. Thus, the suppression of this metabolite by probiotic supplementation may result in a reduction of the precursors to cardiovascular disease.

#### 3.4.3. Probiotic-Induced Reversion in the Kidney

The kidneys of the higher-fat cohort of pigs were devoid of indoxyl sulfate. As previously stated, indoxyl sulfate is a byproduct of protein putrefaction (converted from indole to indoxyl sulfate in the liver and then excreted through the kidneys) [52,53]. Probiotic supplementation of pigs fed the higher-fat diet reverts the level of indoxyl sulfate to that observed within the basal diet, resulting in an appearance of this metabolite in three of the five pigs. Although the exact reason for the resurgence of indoxyl sulfate is unclear, we can speculate that due to the abundance of probiotic bacteria within the GI tract, protein putrefaction may be reestablished even though the pigs are still consuming a higher-fat diet [96]. Further evidence is needed to substantiate this claim.

## 4. Conclusions

In this investigation we explored the effects of a higher-fat diet, and the influence dietary probiotic supplementation has on metabolism. Based on the results presented here, we conclude that a change in diet alone has a profound effect on the tissue metabolome. Additionally, a higher-fat diet has an impact on the tissues examined in this investigation, with the kidney and brain cortex appearing to be the most sensitive. With respect to a higher-fat diet and in agreement with our previous results, these alterations correlate to impaired liver function, increased bile synthesis and purine degradation, and reduced detoxification. Further, this study demonstrates how probiotic supplementation can also influence the metabolome composition. Notably, the metabolic differences identified by probiotic supplementation include alterations to metabolic flux through the hexosamine biosynthetic pathway, thereby reducing intracellular UDP-GlcNAc levels. Thus, suggesting an altered abundance in glycoprotein production and (when coupled to an obseogenic diet) possibly preventing metabolic syndrome. Additionally, probiotic supplementation to the nutritionally balanced basal diet alters lysine catabolism, presumably reflecting preferential lysine utilization by the microbes within the GI tract.

Finally, through an in-depth analysis of the basal, higher-fat, and higher-fat+probiotic cohorts, we discovered that with probiotic supplementation, some potentially aberrant metabolites identified in the higher-fat cohort tissues (notably, S-adenosylhomocysteine and glutathione in the brain cortex, indoxyl sulfate in the kidney, and LysoPE (18:2) in the heart) revert to levels associated with those found in pigs fed a healthy, nutritionally balanced diet. While it remains unknown as to why these higher-fat altered metabolites are particularly sensitive to probiotic supplementation, we can speculate that their reversion to a healthy-associated metabotype may promote healthy neurocognitive function, clear toxic by-products of protein putrefaction, and reduce hallmarks of atherosclerosis [28,29,30,31,36,37,52,53,90,91,92,93,94,95,96]. Coupled together, the reversion of the intracellular concentration of these metabolites to a level akin to a healthy diet depicts the beneficial nature of probiotic supplementation and may show its usefulness as a prophylactic approach to address obesity and metabolic syndrome.

## Figures and Tables

**Table 1:** Top MS/MS-identified metabolites, exhibiting the greatest degree of change in all tissues when comparing basal versus higher-fat diets.

Tissue	Metabolite Name	log_2_(Fold Change)	−log_10_(*p*-Value)	Frequency: Basal (n = 5)	Frequency: Higher-Fat (n = 5)
Brain Cortex	S-adenosylhomocysteine	18.99	1.77	0	3
Brain Cortex	Glutathione	−21.95	1.90	4	0
Heart	Arabitol	−17.62	2.12	3	0
Heart	Betaine	−17.19	1.45	4	0
Kidney	1-(Octadecenoyl)-sn-glycero-3-phosphoethanolamine	15.31	2.27	0	3
Kidney	Indoxyl sulfate	−17.57	1.35	3	0
Liver	1-(Octadecenoyl)-sn-glycero-3-phosphoethanolamine	14.88	2.06	0	4
Liver	Glycocholic acid	4.73	1.66	1	5
Liver	1-O-Hexadecyl-sn-glycero-3-phosphocholine	−17.12	1.92	4	0
Skeletal Muscle	Inosine	19.55	1.64	0	4
Skeletal Muscle	Uridine diphosphate glucuronic acid	15.00	1.65	0	4
Skeletal Muscle	7α-Hydroxy-3-oxo-4-cholestenoic acid	1.85	1.81	4	5
Skeletal Muscle	1-(Octadecenoyl)-sn-glycero-3-phosphoethanolamine	1.83	1.46	1	5
Pancreas	1-(Octadecenoyl)-sn-glycero-3-phosphoethanolamine	2.38	1.42	1	5
Pancreas	Trimethylamine N-oxide	−2.64	1.54	5	4
Pancreas	Betaine	−2.45	1.85	5	3

Criteria for Selection: Frequency ≥ 60% in any one cohort; −log_10_(*p*-value) ≥ 1.3; |log_2_(Fold-Change)**|** ≥ 1.5; Median abundance values ≥ 25,000. Note: a positive fold-change when comparing a basal diet versus a higher-fat diet denotes an increase in abundance for the higher-fat cohort. n equals the number of samples in the cohort in which that metabolite was identified. Features were putatively identified via MS/MS.

**Table 2:** Top metabolites exhibiting the greatest degree of change in all tissues, when comparing basal versus basal+probiotic and higher-fat versus higher-fat+probiotic.

Dietary Comparisions						
Basal versus Basal+probiotic	**Tissue**	**Metabolite Name**	**log_2_(Fold Change)**	**−log_10_(*p*-Value)**	**Frequency: Basal** **(n = 5)**	**Frequency:** **Basal+Probiotic** **(n = 5)**
	Brain Cortex	Phosphatidylethanolamine lyso alkenyl 18:1	15.79	3.13	3	0
	Brain Cortex	1-Octadecanoyl-sn-glycero-3-phosphocholine	3.28	1.39	3	3
	Brain Cortex	Taurine	−18.63	7.75	0	3
	Heart	6-amino-2-[[3-methyl-2-[[pyrrolidine-2-carbonyl]amino]butanoyl]amino]hexanoic acid	14.90	2.02	4	0
	Heart	(4-amino-4,6-dimethyl-5-sulfooxy-tetrahydropyran-2-yl) [hydroxy-[[3-hydroxy-5-(5-methyl-2,4-dioxo-pyrimidin-1-yl)tetrahydrofuran-2-yl]methoxy]phosphoryl] hydrogen phosphate	1.69	1.32	5	3
	Heart	tert-butyl N-[1-[[1-cyclohexyl-2-hydroxy-2-[6-(phenylcarbamoyl)-3,3a,4,5,6,6a-hexahydro-2H-pyrrolo [3,2-b]pyrrol-1-yl]ethyl]amino]-1-oxopropan-2-yl]-N-methylcarbamate	−15.54	3.10	0	4
	Kidney	Uridine diphosphate-N-acetylglucosamine *	22.1	4.62	4	0
	Kidney	Urothion	15.62	4.40	4	0
	Kidney	Uridine 5′-diphosphogalactose	−14.86	6.61	0	3
	Liver	Saccharopine *	16.94	4.51	4	0
	Liver	LysoPC(18:0)	15.81	4.14	3	0
	Liver	[5-(2,6-dihydroxy-2,3-dihydropurin-9-yl)-3,4-dihydroxy-tetrahydrofuran-2-yl]methyl phosphono hydrogen phosphate	−18.17	3.34	0	3
	Skeletal Muscle	1-O-Hexadecyl-2-O-acetyl-sn-glyceryl-3-phosphorylcholine	20.67	2.86	3	0
	Skeletal Muscle	4-Hydroxy-3-methoxymandelic acid	15.14	3.35	3	0
	Skeletal Muscle	Uridine 5′-diphosphogalactose	−15.27	1.75	0	3
	Pancreas	N-(2-aminoacetyl)-1-(4-phenyldiazenylphenyl)pyrrolidine-2-carboxamide	18.39	4.39	3	0
	Pancreas	4-hydroxypyrrolidine-2-carbaldehyde	−6.04	2.69	5	5
	Pancreas	Guanosine diphosphate mannose	−15.39	3.52	0	3
Higher-fat versus Higher-fat+probiotic	**Tissue**	**Metabolite Name**	**log_2_(Fold Change)**	**−log_10_(*p*-value)**	**Frequency:** **Higher-fat** **(n = 5)**	**Frequency:** **Higher-fat+probiotic** **(n = 5)**
	Brain Cortex	S-adenosylhomocysteine *	18.99	3.74	3	0
	Brain Cortex	1-Stearoyl-2-hydroxy-sn-glycero-3-phosphocholine	18.39	1.40	3	0
	Brain Cortex	5,6,7,8-Tetrahydromethanopterin	−18.71	5.05	0	3
	Heart	2′-α-mannosyl-L-tryptophan	15.31	6.63	3	0
	Heart	N’-{[8-({5-[2-(2-aminopyridin-4-yl)ethyl]-3,4,5-trihydroxy-6-(hydroxymethyl)oxan-2-yl}oxy)-1-hydroxy-5-(hydroxymethyl)-6-methoxy-9,10-dioxo-3-(2-phenylethyl)-9,10-dihydroanthracen-2-yl]methyl}-N-methylguanidine	15.56	7.09	3	0
	Heart	3,4,5-Trinitrofuran-2-thiol	−16.58	3.45	0	3
	Kidney	Uridine diphosphate-N-acetylglucosamine *	22.45	8.07	3	0
	Kidney	3-sulfolactic acid	−16.66	3.92	0	3
	Kidney	Indoxyl sulfate *	−19.18	2.79	0	3
	Liver	Adenosine monophosphate	20.96	2.21	3	0
	Liver	Phosphodimethylethanolamine	−19.45	3.73	0	4
	Liver	6-(7-hydroxy-4,6-dimethylhepta-2,4-dien-2-yl)-4-methoxy-5-methyl-2H-pyran-2-one	−22.02	5.30	0	4
	Skeletal Muscle	Inosine *	19.55	2.79	4	0
	Skeletal Muscle	Xylitol	16.88	4.37	4	0
	Skeletal Muscle	2-[[5-acetamido-6-(2-amino-2-carboxy-1-methyl-ethoxy)-3,4-dihydroxy-tetrahydropyran-2-yl]methoxy]-4-hydroxy-5-[[2-(2-methoxyethoxycarbonylamino)acetyl]amino]-6-(1,2,3-trihydroxypropyl)tetrahydropyran-2-carboxylic acid	−14.75	4.02	0	3
	Pancreas	LysoPE(20:1)	20.76	2.94	4	0
	Pancreas	Guanosine monophosphate	17.74	5.19	3	0
	Pancreas	N-({6-[(5-tert-butyl-1,2-oxazol-3-yl)methyl]-3-hydroxyoxan-2-yl}methyl)propanamide	−17.82	4.60	0	3

Criteria for Selection: Frequency ≥ 60%; •log_10_(*p*-value) ≥ 1.3; |log_2_(Fold-Change)**|** ≥ 1.5; Median abundance values ≥ 25,000. Note: a positive fold-change when comparing basal diets versus basal+probiotic indicates greater abundance in the basal cohort. A positive fold-change when comparing higher-fat diets versus higher-fat+probiotic denotes greater abundance in the higher-fat cohort. n equals the number of samples in the cohort. * Features were identified via MS/MS. All other features were identified using the formula proposed by MS-FINDER.

**Table 3:** Metabolites associated with probiotic-induced reversion of the higher-fat metabolome.

Tissue	Metabolite Name	log_2_(Fold Change) Higher-Fat vs. Basal	log_2_(Fold-Change) Higher-Fat+Probiotic vs. Basal	Frequency: Higher-Fat (n = 5)	Frequency: Basal (n = 5)	Frequency: Higher-Fat+Probiotic (n = 5)
Brain Cortex	S-Adenosylhomocysteine *	19.00	0.00	3	0	0
Brain Cortex	Tryptophan	4.58	−0.24	3	3	4
Brain Cortex	4-(Methylsulfanyl)-2-oxobutanoic acid	3.91	−0.46	3	3	3
Brain Cortex	LysoPC(22:4)	−14.06	0.04	0	4	3
Brain Cortex	2-[[2-(hexadecanoylamino)acetyl]amino]-3-(1H-imidazol-5-yl)propanoic acid	−14.67	0.01	0	3	3
Brain Cortex	LysoPC(22:6)	−21.26	−0.21	0	3	3
Brain Cortex	Glutathione *	−21.95	0.16	0	4	2
Heart	LysoPE(18:2)	14.36	0.00	4	0	0
Kidney	LysoPC(16:1)	2.04	−0.19	3	2	3
Kidney	Indoxyl sulfate *	−17.57	1.61	0	3	3

Criteria for Selection: Frequency ≥ 60%; *p* value < 0.05; |log_2_(Fold-Change)| ≥ 2 in higher-fat vs. basal AND |log_2_(Fold Change)| ≤ 2 in higher-fat+probiotic vs. basal; Median abundance values ≥ 25,000. n is equal to the number of samples in the cohort. Note: a positive fold-change when comparing higher-fat versus basal diets indicates an increase in abundance for higher-fat pigs. A positive fold-change when comparing higher-fat+probiotic versus basal diets indicates an increase in abundance for the higher-fat+probiotic cohort. * Features were identified via MS/MS. All other features were identified using the formula proposed by MS-FINDER.

## Data Availability

The datasets generated during and/or analyzed during the study are available from the corresponding author on request. Data is not publicly available due to privacy.
